# New insights into culture negative endophthalmitis by unbiased next generation sequencing

**DOI:** 10.1038/s41598-018-37502-w

**Published:** 2019-01-29

**Authors:** Dhanshree Deshmukh, Joveeta Joseph, Moumita Chakrabarti, Savitri Sharma, Rajagopalaboopathi Jayasudha, Kalyana C. Sama, Bhavani Sontam, Mudit Tyagi, Raja Narayanan, S. Shivaji

**Affiliations:** 1 Jhaveri Microbiology Centre, Brien Holden Eye Research Centre, L. V. Prasad Eye Institute, Hyderabad, Telangana India; 2 Smt. Kannuri Santhamma Centre for vitreoretinal diseases, L V Prasad Eye Institute, Hyderabad, India

## Abstract

The proof-of-concept, study to investigate the presence of microorganisms in presumed infectious endophthalmitis using Next generation sequencing (NGS) was carried out in vitreous biopsies from 34 patients with endophthalmitis, and thirty patients undergoing surgery for non-infectious retinal disorders as controls. Following DNA extraction using the Qiagen mini kit and PCR amplification of the V3–V4 regions of the bacterial 16S rRNA and ITS 2 region of fungus, they samples were sequenced on an Illumina HiSeq 2500 Machine. Paired reads were curated, taxonomically labeled, and filtered. Culture based diagnosis was achieved in 15/34 (44%) patients while NGS diagnosed the presence of microbes in 30/34 (88%) patients (bacteria in 26/30, fungi in 2/30, mixed infections in 2/30 cases). All 30 controls were negative for bacteria or fungus by NGS. There was good agreement between culture and NGS for culture-positive cases. Among culture negative cases, DNA of common culturable bacteria were identified like *Streptococcus* sp., *Staphylococcus* sp., *Pseudomonas* sp., *Gemella* sp., *Haemophilus* sp., *Acinetobacter sp*. The specificity of NGS with culture and clinical diagnosis was found to be 20% and 100% respectively and sensitivity of NGS with culture and clinical diagnosis was found to be 87.5% and 88% respectively. NGS appears to be promising diagnostic platform for the diagnosis of infectious culture negative endophthalmitis.

## Introduction

Endophthalmitis, is a potentially sight-threatening condition that varies geographically in incidence and in cause, following surgical procedures, trauma or endogenous dissemination^1^. The ability to identify the causative organism has huge implications in treatment and clinical management of the patients. Extensive variation in culture positivity from 38–44% in clinically diagnosed endophthalmitis cases is known and has been reported in Indian subjects^2–4^. The aetiologic agent is therefore unknown in a majority of patients, when routine aerobic bacterial cultures are negative. Culture-negativity could be attributed to fastidious nature of the inciting organism that may be difficult to grow in culture or may be unculturable. Inspite of the limitation of low yield and inability of certain pathogens to grow on routine media, microbiological culture still remains the current gold standard for the diagnosis of most intraocular infections. The use of molecular tools like PCR has improved the yield of detection in addition to reducing the time to make a confirmatory diagnosis^5^. However the number of fungal and/or bacterial pathogens that can be simultaneously detected is limited, due to differences in amplification efficiencies of different primer sets and the limited number of fluorescent labels because of which multiplexing different PCRs assays becomes technically challenging.

In comparison, Next-generation sequencing (NGS) is assumption-free, meaning that it does not target just one specific species but can detect all the different fungi/bacteria present in a clinical sample in one single assay. This technique promises not only improved detection of traditional organisms but can also has the potential to identify newer organisms not previously associated with endophthalmitis. Recent reports of the presence of Torque Teno Virus in the vitreous of patients diagnosed with endophthalmitis has substantiated this claim^6^. We would like to test the feasibility of the application of next generation DNA sequencing to vitreous biopsies from patients with endophthalmitis and compare the results with traditional culture techniques. Accurate diagnosis of endophthalmitis using next generation sequencing may not only makes it feasible to determine a better treatment startegy in these cases, but it may also improve outcome in culture negative cases in which delayed diagnosis has likely contributed to historically poor outcomes and may become the new standard in the management of intraocular infections.

## Results

A total of 34 presumed infectious endophthalmitis – aqueous/vitreous biopsies and 30 vitreous control samples were included in the study. There were 22 males and 12 females in the test group and the mean age was 35 ± 25.6 years. Clinical and demographic details of patients are given in Table 1. The most common risk factor linked with presumed infectious endophthalmitis was trauma in 21 (61.7%) patients followed by cataract surgery in 7 patients (20.5%), endogenous source in 2 cases, non resolving fungal keratitis in 2 cases and in remaining 2 cases it was unknown. Initially patients were treated with injection of intravitreal antibiotics (vancomycin 1 mg/0.1 ml and ceftazidime 2.25 mg/0.1 ml) with or without intravitreal dexamethasone (400 μg/0.1 ml) in addition to pars plana vitrectomy (PPV) in 25 (73.5%) patients and/or lensectomy and vitrectomy along with intravitreal antibiotics in 9 (26.4%) of the 34 patients. In 3 patients there was a clinical suspicion of fungal infection and additional intravitreal amphotericin B (5 µg/0.1 ml) was given while one patient (#25) was given intravitreal and intracameral linezolid as it was a suspected case of *Pythium* keratits. Two patients had an intraocular foreign body removal (IOFB-R) while one required intraocular lens (IOL) to be explanted. The medical treatment also included intensive mainly ciprofloxacin (0.3% 1 hourly) and prednisolone acetate (1% one hourly) along with oral ciprofloxacin 750 mg (2/day for 7–10 days). The 5 year-old child (#29) was given cephalexin syrup (125 mg twice daily × 6 days) and intravitreal gentamicin. None of the patients were clinically immunocompromised. However, we had two patients with endogenous endophthalmitis who were children aged one and six years and it is possible that prior to reporting to our hospital with endogenous endophthalmitis the children were ill, the details of whch were not available to us. Post-treatment 7/34 (20.5%) patients had a visual outcome of 20/200 or better.

**Table 1:** Clinical and Demographic details of the patients with presumed Infectious Endophthalmitis included in the study.

Sample ID	Age	Sex	Diagnosis	Predisposing factor	Duration	Initial VA	Surgery	Treatment	Final VA	Microbiology culture
1	71	F	Traumatic Endophthalmitis	Injury - thorn	10days	HM+	PPV	V + C	NPL	No growth
2	56	F	Early Endophthalmitis	Microbial Keratitis	3 days	HM+	Vit Bx	V + C	HM+	No growth
3	28	M	Traumatic Endophthalmitis	Injury - safety pin	7 days	HM+	AC Wash* + vit Bx	V + C	20/320	*Streptococcus mitis*
4	7	M	Traumatic Endophthalmitis	Injury - wooden stick	10 days	20/800	PPL + PPV	(V + C) + (C + Dexa)	20/80p	*Stenotrophomonas maltophila*
5	30	M	Traumatic Endophthalmitis	Injury - iron rod	1 day	LP + PR+	PPV	V + C	CFCF	No growth
6	14	M	Traumatic Endophthalmitis	Injury - stick	7 days	LP + PR −	PPL + PPV	V + C + Amp B	HM+	*Scedosporium apiospermum*
7	7	M	Delayed Endophthalmitis	Cat Sx - 10 months	4 days	HM+	PPV	V + C	CFCF	No growth
8	37	M	Traumatic Endophthalmitis	Injury - thorn	2 days	LP + PR+	PPV + PPL	V + C	20/40	No growth
9	7	M	Traumatic Endophthalmitis	Injury - stick	3 days	LP + PR−	PPL + PPV	V + Dx	phthisis	*Streptococcus pneumoniae*
10	34	M	Traumatic Endophthalmitis	Injury - accident	4 days	HM+	PPV	V + C	20/25	*S*. *epidermidis*
11	66	M	Traumatic Endophthalmitis	Injury - wooden piece	4 days	20/320	PPV + SICS	V + C	20/200	No growth
12	51	M	Traumatic Endophthalmitis	Injury - thorn	10 days	HM+	AC TAP + PPV	V + C	20/100	No growth
13	14	M	Traumatic Endophthalmitis	Injury - thorn	3 days		HM + CTR + PPV + IOFB	V + C	20/125p	*Pantoea sp*.
14	7	F	Acute Endophthalmitis	—	5 days	LP + PR −	PPV	V + C + Dx	NPL	*S*. *epidermidis*
15	1	F	Endogenous Endophthalmitis	—	1 week	LP + PR+	PPL + PPV	V + C	Not cooperative	*S*. *pneumoniae*
16	8	M	Traumatic Endophthalmitis	Injury - finger	4 days	LP + PR −	PPV	V + C	LP + PR −	No growth
17	61	F	Post operative Endophthalmitis	Cat Sx	51 days	CF CF	PPV + IOL explant	V + C	20/40	No gr
18	41	F	Traumatic Endophthalmitis	Injury - wooden stick	1 day	20/320	PPV	V + C	20/125	No growth
19	43	F	Traumatic Endophthalmitis	Injury	30 days	HM+	PPL + PPV	V + C	20/125	*Streptococcus pseudoporcinus*
20	71	M	Early Endophthalmitis	Non resolving FUNGAL KERATITIS	7 days	HM+	Vit Bx	V + Amp B	phthisis	*Streptococcus pneumoniae*
21	5	M	Traumatic Endophthalmitis	Injury - needle	5 days	HM+	PPV + MP + SOI	V + C + Dx	phthisis	*Bacillus sp*., *Staphylococcus hominis*, *Enterobacter cloacae*, *Streptococcus sp*.
22	10	F	Traumatic Endophthalmitis	Injury - plastic band	5 days	NPL	PPL + PPV	V + C	20/60	No growth
23	41	M	Traumatic Endophthalmitis	Injury - stick	5 days	LP + PR+	PPV	V + C + Dx	20/60	*Staphylococcus hominis*
24	14	M	Traumatic Endophthalmitis	Injury - wire	3 days	HM + PPL+	PPV	V + C	phthisis	No growth
25	49	M	Early Endophthalmitis	Fungal keratitis	10 days	LP + PR −	PPV	intravit and intraca- meral linezolid	LP + PR −	No growth
26	41	F	Post surgical Endophthalmitis	Cat Sx - 1 months	28 days	LP + PR+	PPV	V + C	20/30	*Achrobacter xylosoxidans*
27	50	M	Traumatic Endophthalmitis	Injury - foreign body	20 days	HM+	PPV + IOFB-R + EL	V + C	20/400	No growth
28	50	M	Post surgical Endophthalmitis	Cat Sx	20 days	LP + PR+	PPV	Amp B	phthisis	No growth
29	5	F	Traumatic Endophthalmitis	Injury - needle	5 days	uncooperative	PPL + PPV+ AC WASH	IV Gen	Not cooperative	*Morganella morganii*
30	56	M	Early Endophthalmitis	Microbial Keratitis	30 days	HM+	Vit Bx	V + C	HM+	No growth
31	61	F	Post surgical Endophthalmitis	Cat Sx - 20 days	3days	LP + PR −	PPV	V + C + Dx	20/125	*Pseudomonas luorescens*
32	8	M	Traumatic Endophthalmitis	Injury - needle	4 days	NPL	PPV	V + C	HM+	No growth
33	76	F	Post surgical Endophthalmitis	Cat Sx	28 days	HM+	PPV	V + C + Dx	HM+	No growth
34	71	M	Post surgical Endophthalmitis	Cat Sx	2 days	LP + PR −	PPV	V + C	CF CF	No growt

Legend- M: male; F: female; Cat Sx: Catract surgery; HM: Hand Movements; LP + PR+: Light perception and accurate projection of rays; LP + PR−: Light perception with inaccurate projection of rays; CFCF: Counting ingers close to face, NPL: No light perception; PPV: pars plana vitrectomy; PPL: pars plana lensectomy; Vit Bx: vitreous biopsy; CTR: Corneal tear repair; IOFB-R: Intraocular foreign body removal; MP: Membrane Peeling; V: Vancomycin; C: Ceftazidime; D Dx: Dexamethasone; Gen: Gentamicin; Amp B: Amphotericin B; AC Tap: Aqueous biopsy.

### Microbiology culture

A total of 15 of the 34 samples were positive by microbial culture (Table 1) of which 14 grew bacteria and one case showed growth of fungal organism. Five samples were positive for *Streptococcus* species while three samples showed presence of *Staphylococcus* sp. The remaining five culture-positive samples grew gram negative bacilli and one showed growth of polymicrobial organisms including *Bacillus* sp. along with *Staphylococcus* and *Streptococcus* species.

### Taxonomic analysis by NGS

**Figure 1:**
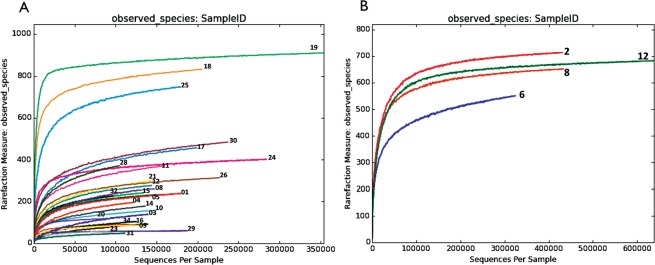
Observed Rarefaction curves calculated using the OTU matrix obtained from the Illumina HiSeq raw data by using QIIME are shown for all the analysed samples and sequencing runs. OTUs diversity for each sample obtained (Left) using V3–V4 primers (Right) ITS2 primers.

The sequence dataset generated by Illumina sequencing of V3–V4 16S rRNA gene amplicons with 20X coverage yielded a total of 4.7 million high-quality reads (mean = 0.17 million reads/sample ± 0.07 million [s.d.]; range = 91735–417693 reads) and clustered into 2,115 OTUs. For fungal ITS data, we could obtain 193, 2010 reads that clustered into1450 OTUs. Rarefaction plots revealed that the sequencing coverage were sufficient for data comparison, as all samples entered the plateau phase (Fig. 1A,B). None of 30 control eyes undergoing routine vitreous surgery yielded positive results for bacteria or fungus by NGS.

**Figure 2:**
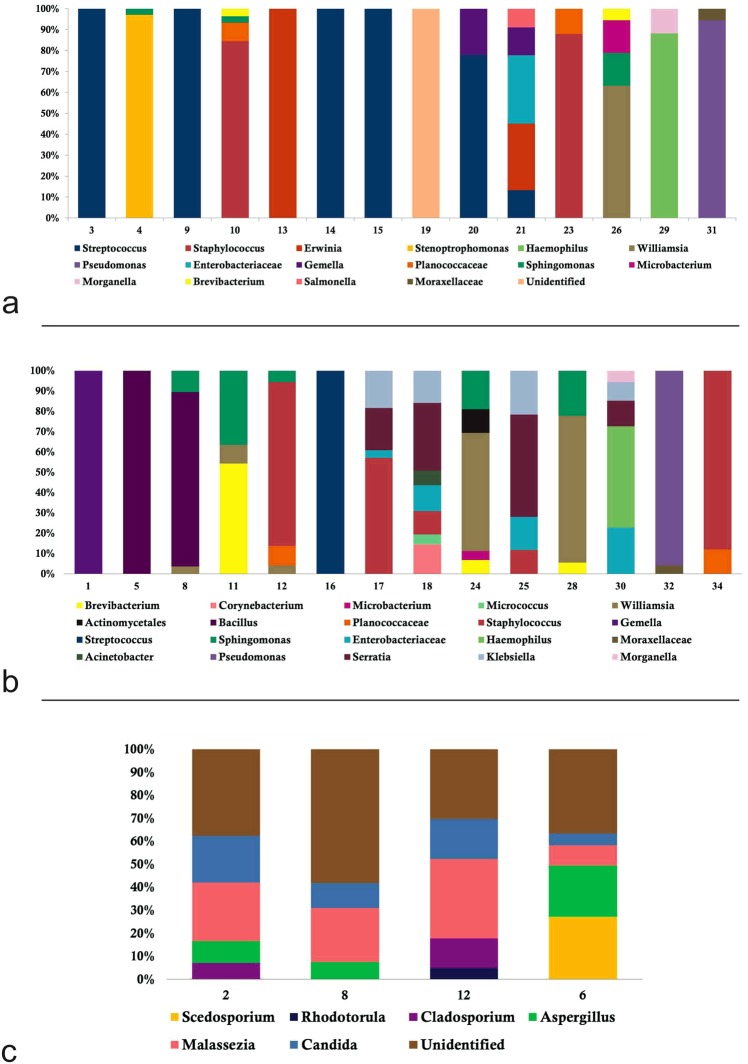
Stacked box-plot displaying the relative abundances of the bacterial taxa (≥3%) and species richness obtained by NGS on vitreous samples of (**a**) Culture positive endophthalmitis (**b**) Culture Negative endophthalmitis (**c**)Stacked box-plot displaying the relative abundances of the fungal taxa (≥3%) and species richness obtained by NGS on vitreous samples of both Culture positive (#2) and Culture Negative samples (#8,12,6).

NGS detected presence of clinically significant bacteria in 28 patients (82.3%), including 14 of the culture positive patients (Fig. 2a) and 14 patients with a negative culture (Fig. 2b). In addition, NGS also detected presence of fungus in 4 patients (pure fungus-2, mixed with bacteria-2) of which one was culture positive and 3 were culture negative (Fig. 2c). Totally, among the culture negative specimens, NGS showed presence of bacteria in 12 cases and fungus in 2 cases (#8 & 12), while 2 showed presence of both bacteria and fungus, taking the total positivity rate to 30/34 (88.2%) patients as shown in Tables 3 and 4. Four out of 34 samples did not contain significant levels of bacterial or fungal DNA hence NGS could not be performed. For all samples, the assay generated reproducible reads upto the genus level mainly and not to the species level. Additionally, NGS suggested presence of polymicrobial organisms in most of the culture-negative and few culture positive samples (Table 2).

**Table 2:** Taxonomic lineage identified in Culture positive cases of Endophthalmitis.

Sample ID	Microbiology Culture	Taxonomic Lineage through NGS
3	*Streptococcus mitis*	*Streptococcus* 100%
4	*Stenotrophomonas maltophila*	*Stenotrophomonas* 91%, *Sphingomonas* 3%
9	*Streptococcus pneumoniae*	*Streptococcus* 100%
10	*S*. *epidermidis*	*Staphylococcus* 83%, Planococcaceae 9%, *Sphingomonas* 3%, *Brevibacterium 4%*
13	*Pantoea* sp.	*Erwinia* 99%
14	*S*. *epidermidis*	*Streptococcus* 100%
15	*S*. *pneumoniae*	*Streptococcus* 98%
19	*Streptococcus pseudoporcinus*	Unidentified 98%
20	*S*. *pneumoniae*	*Streptococcus* 78%, *Gemella* 22%
21	*Bacillus* sp., *Staphylococcus hominis*, *Enterobacter cloacae*, *Streptococcus* sp.	*Streptococcus* 12%, *Erwinia* 28%, Enterobacteriaceae 29%, *Gemella* 12%, *Salmonella* 8%
23	*Staphylococcus hominis*	*Staphylococcus* 88%, Planococcaceae 12%
26	*Achrobacter xylosoxidans*	*Williamsia* 56%, *Sphingomonas* 14%, *Microbacterium* 14%, *Brevibacterium* 5%
29	*Morganella morganii*	*Haemophilus* 87%, *Morganella* 12%
31	*Pseudomonas fluorescens*	*Pseudomonas* 94%, Moraxellaceae 6%

**Table 3:** NGS reads and Taxonomic lineage identified in Culture negative cases of Endophthalmitis.

Sample ID	Taxonomic Lineage through NGS
1	*Gemella* – 95%
5	*Bacillus* – 96%
8	*Bacillus* – 81%, *Sphingomonas* – 10%, *Williamsia* – 3%
11	*Brevibacterium* – 34%, *Sphingomonas* – 23%, *Williamsia* – 6%
12	*Staphylococcus* – 70%, Planococcaceae – 8%, *Sphingomonas* – 5%, *Williamsia* – 4%
16	*Streptococcus* – 100%
17	*Staphylococcus* – 47%, *Serratia* – 17%, *Klebsiella* – 15%, Enterobacteriaceae – 3%
18	*Serratia* – 21%, *Klebsiella* – 10%, *Corynebacterium* – 9%, Enterobacteriaceae – 8%, *Staphylococcus* – 7%, *Acinetobacter* – 4%, *Micrococcus* – 3%
24	*Williamsia* – 42%, *Sphingomonas* – 14%, Actinomycetales – 9%, *Brevibacterium* – 5%, *Microbacterium* – 3%
25	*Serratia* – 37%, *Klebsiella* – 16%, Enterobacteriaceae – 12%, *Staphylococcus* – 9%
28	*Williamsia* – 61%, *Sphingomonas* – 19%, *Brevibacterium* – 5%
30	*Haemophilus* – 44%, Enterobacteriaceae – 20%, *Serratia* – 11%, *Klebsiella* – 8%, *Morganella* – 5%
32	*Pseudomonas* – 91%, Moraxellaceae – 4%
34	*Staphylococcus* – 87%, Planococcaceae – 12%

**Table 4:** NGS reads and taxonomic lineage of fungi identified in presumed Endophthalmitis cases in the study.

Sample ID	Microbiology Culture	Taxonomic Lineage through NGS
2	—	Unidentified 33.51%, *Malassezia* 22.85%, *Candida* 18.2%, *Aspergillus* 8.43%, *Cladosporium* 6.42%
8	—	Unidentified 49.42%, *Malassezia* 19.97%, *Candida* 9.35%, *Aspergillus* 6.36%
12	—	*Malassezia* 27.56%, Unidentified 24.21%, *Candida* 13.9%, *Cladosporium* 10.14%, *Rhodotorula* 4.05%
6	*Scedosporium apiospermum*	Unidentified 32.75%, *Scedosporium* 24.35%, *Aspergillus* 19.93%, *Malassezia* 7.93%, *Cladosporium* 4.58%

### Concordance between culture and NGS techniques for culture positive endophthalmitis

There was good agreement among culture, and NGS for culture-positive cases as shown in Table 2. All the 14 bacterial culture positive cases showed the presence of DNA of the same organism by NGS along with simultaneous presence of other species of bacteria and/or fungus (Table 2). In 12/14 cases, the bacteria that grew in culture was also identified by NGS (>80%) which included monobacterial infection in two cases of *Streptococcus* sp. infection, one case of *Erwinia* sp. infection (previously *Pantoea* sp.), as shown in Fig. 2a. One case (#29) grew *Morganella* sp. in culture, however NGS showed the sample to have predominantly DNA of *Haemophilus* species (87%) and only 12% of reads of *Morganella sp*. was present. Similarly one case which grew *Staphylococcus epidermidis* in culture (#14), while in NGS *Streptococcus* sp. was present and in other case which grew *Achrobacter xylosoxidans* in culture (#26), however NGS showed the sample to have DNA of *Williamsia sp*. 56%, *Sphingomonas sp*. 14%, *Microbacterium sp*. 14%, *Brevibacterium sp*. 5% (Fig. 2a). In 2/14 cases there was a complete mismatch of the bacteria that grew in culture with the DNA that was identified in NGS. Similarly the one case that showed growth of *Scedosporium apiospermum* in fungal culture, was present in only 2.9% of the reads by NGS, while predominantly *Aspergillus sp*., *Candida* sp. and unidentified fungus DNA was identified by NGS (Fig. 2c).

### NGS analysis for culture negative endophthalmitis

Among the 14/19 culture negative endophthalmitis cases included in the study that showed presence of DNA of bacterial pathogens, only 3/14 were mono bacterial while the rest showed presence of polybacterial infections, as shown in Table 3. In addition DNA of common culturable bacteria that were also present in culture positive endophthalmitis like *Streptococcus sp*., *Staphylococcus sp*., *Pseudomonas sp*., *Gemella sp*. were also detected as shown in Fig. 2b. The presence of fungal DNA in three culture negative samples was also predominantly that of unidentified species, followed by *Candida sp*., *Aspergillus sp*., *Malassezia sp*., *Scedosporium sp*., *Rhodotorulla sp*. (Fig. 2c). The presence of several unknown fungal taxonomic groups indicates the limitations of the existing fungal sequence databases (Table 4).

**Figure 3:**
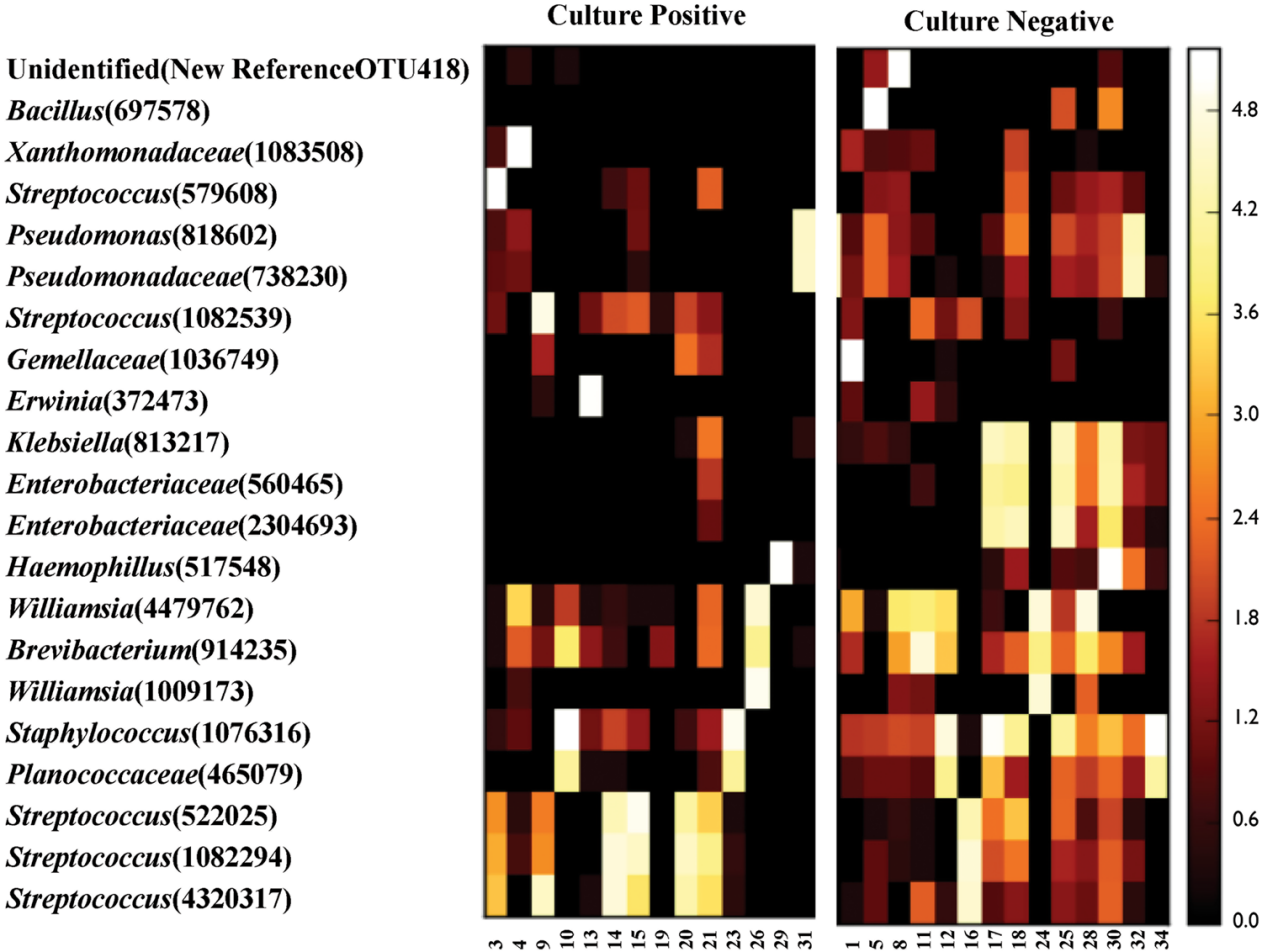
Heat map of Genera Level Bacterial Abundances in samples from patients with presumed infectious both culture positive and culture negative endophthalmitis. The color bar on the right side indicates the average relative abundances of these genera in each patient.

Combining the NGS results by generating a Heat map of all samples showed that the taxonomic abundance for bacterial species detected in 28 samples (including culture positive and culture negative) of presumed infectious endophthalmitis was predominantly of gram positive species as shown in (Fig. 3).

### Statistical analysis

The sensitivity and specificity of NGS with culture as a diagnostic tool was 87.5% and 20%, however, when we compared NGS with clinical diagnosis, the sensitivity and specificity improved to 88% and 100% respectively. Additionally no bacterial genera were found to be significantly differentially enriched in both culture positive and culture negative endophthalmitis cases by Mann-Whitney- Wilcoxon test after post-hoc correction (Supplementary Table 3).

## Discussion

We report the outcome of a proof-of-concept study that uses next generation sequencing on vitreous fluid for the identification of bacteria and fungi in patients with presumed infectious endophthalmitis as well as to understand the genesis and surreptitiousness of culture-negative endophthalmitis. Consistent with previous results, approximately 50–60% of the cases included in the study were culture-negative. Although the sample size in our study was small, we showed that culture-negative cases of clinical endophthalmitis are not devoid of organisms and predominantly gram positive organisms dominate the microbial load. As NGS is entirely based on bacterial DNA detection as against the viable organisms in culture, the etiologic basis of culture-negative endophthalmitis still remains unclear. Possibilities for this condition include, presence of fastidious organisms and prior antimicrobial therapy^7^ that may inhibit microbial growth during culture or infection with non-bacterial pathogens to scant (undetectable) bacterial pathogens, or true ‘sterile’ endophthalmitis associated with antigenic response to a non-infectious antigen. In such patients where the culture results have been compromised by prior healthcare exposure that NGS has the potential to increase the diagnostic yield for detection of microbes compared to current methods especially in a tropical country like ours, where the probablility of fungal endophthalmitis is high. Though the antimicrobial susceptibility can not be performed on pathogens identified by this procedure, we believe that the knowledge of published literature on the susceptibility profile of the organisms may help determine inclusion of an effective antibiotic if required for the management. For example, modification in the treatment of mycobacterial endophthalmitis would require addition of rifampicin/amikacin to the current treatment protocol. Similar to previous studies, while we find concordance between NGS results and culture results for culture-positive cases^6,8,9^. Unlike previous studies, however, the author find the majority of culture-negative results being associated with bacterial and/or fungal pathogens at the NGS level^6^. To ensure that the NGS results were not due to contaminating DNA and/or due to technical errors appropriate reagent controls were run with every PCR reaction to be able to detect contaminating DNA. Our results were consistently negative for DNA from all reagents including DNA extraction kits, PCR reagents etc. Validation of NGS requires the calculation of analytical sensitivity and specificity, and comparing with culture it was found to be 87.5% and 20% respectively. However, this standard method of calculation is probably not appropriate in this study as our focus is on culture negative cases and it defeats the rationale of taking culture as gold standard. So taking clinical diagnosis of endophthalmitis as gold standard, the sensitivity and specificity of NGS was found to be 88.0% and 100% respectively.

Though molecular methods overcome the limitations of traditional culture, PCR assays suffer from its limited ability to distinguish between contaminations, and true infections, often revealing ambiguous results. In contrast, NGS-based diagnostic testing offers several advantages including providing the opportunity to detect bacterial, fungal, and viral pathogens in a single assay and NGS being quantitative, counting of sequence reads helps in determining the statistical significance. However, although NGS is becoming increasingly important in clinical microbiology, only occasional reports^6,8,9^ of NGS-analyzed ocular specimens have been published to date and our study is among the largest series on its application for clinical diagnostics. The recovery of organisms from culturally sterile vitreous of patients in this study is in presence of clinical inflammation and is in all probability points at a disease entity associated with the organism. This probability is strengthened by the fact that the organisms were not found in control non-infective conditions studied. In addition, our series includes many cases of post-traumatic endophthalmitis which is probably an added strength of our paper.

The NGS assay in our study helped in detection more than one bacterial species in almost all of the confirmed endophthalmitis patients, a diagnosis which was completely missed by culture. It is difficult to explain why some of the organisms were not recovered in culture. Competition among the organisms, fastidious nature, difference in rate of growth, quorum sensing etc. are some of the plausible reasons. Polymicrobial eye infections present a challenge not only in terms of identification of the causative organism, but also in instituting appropriate antimicrobial therapy. Large studies have reported variable prevalence of polymicrobial infection (3.88–20.4% of culture proven endophthalmitis) from different parts of the world including India^2,10^ NGS approach has higher sensitivity in identification of microorganisms present at low abundance than 16S rDNA and ITS PCR library method. So, we believe that the NGS approach would have better sensitivity in culture negative cases (where the load is believed to be low). Additionally, in case of polymicrobial infections, ambiguous electropherograms would hinder identification of bacteria or fungus. Currently, the cost of NGS method is less than regular Sanger sequencing with the library approach method, which is needed when polymicrobial organisms are suspected. The power of NGS is that it provides an assumption-free approach to identify multiple bacteria in a single run thus saving cost.

Since a broad range of bacteria can be detected using NGS, the specificity of the assay is limited and hence it is essential to remove all the background DNA. While, our NGS data analysis workflow included additional filtering steps to reduce false positives due to contaminant DNA sequences in human host and environmental background DNA sequences introduced by our reagents. We did not identify any specific microorganisms in the quality control vitreous samples that are collected at the same time and location as the index samples, suggesting that vitreous fluid is a sterile body part or only contains few microbial cells in individuals without eye infections. Additionally, the NGS used in this study was confined to a short sequence read length of 450–600 bp that reliably allowed only genus level identification, although species could be identified. As far as the treatment of endophthalmitis is concerned, genus level identification is normally adequate, which the NGS of culture negative vitreous samples could achieve. The importance of NGS lies in the fact that it could detect organisms that would have otherwise not been detected in the patients with clinically presumed endophthalmitis. While species level identification by whole genome sequencing is possible, it would be cost prohibitive and hence to reduce costs we feel that a targeted based metagenomics would be useful in a clinical setting.

Further development is required to improve the workflow for NGS, in particular to reduce the turnaround time and costs, in addition to streamlining downstream data analyses. Only when these processes reach maturity will NGS be feasible for routine patient management, thereby enabling the transformation of clinical microbiology into a genome-based and personalized diagnostic field. In most cases the turn around time of culture will be equivalent to a complete NGS analyses. In this context, we would like to add that this is an emerging technology domain and still mostly practiced in research laboratory settings with a turnaround time of around 4–5 days. However, in an optimized setting, if we consider the actual time required for DNA sequencing for a small number of samples, say ~5 hrs on Illumina Miseq, added to the time taken in the lab for sample preparation (DNA extraction + PCR amplification of 16S rDNA regions) and time taken for taxonomic analyses of a small number of samples on a modern-day computer workstation, the total time from sample collection to diagnosis should not take more than 24–48 hours. And we believe that once NGS becomes a routine clinical test, a 48 hr turn around time could become a reality. At present, the Unit costs for current diagnostic microbiological culture on an array of media would be ~USD 25 per sample. Comparatively, the cost of next-generation sequencing for metagenomic testing is high (~USD 90 per sample). However, we only examined the impact of introducing the NGS to the overall diagnostic cost and excluded costs associated with surgical management especially in a scenario of culture negative endophthalmitis. The high cost of metagenomic test may be justified as it provides faster actionable results thereby reducing the length of consulations and hospital visits, which can return significant cost savings. For some patients, a more conclusive diagnosis and shorter turnaround time provided by NGS compared to traditional culture testing may be a premium worth paying for. From a cost perspective, NGS may help to avert second- or third-line investigations and therefore, will most likely be used as an adjunct investigation, when the opportunity for cost savings is greatly diminished.

There are however some limitations in our study, including the limited volume of vitreous fluid available for metagenomics which might have hindered accurate detection of bacteria. Secondly, some species have highly similar 16S rRNA genes and the distinction of closely related species based on the V3-V4 region could be unreliable. The impact on treatment strategies and outcome remains to be seen and we are in the process of using an elaborate study design to decipher the same. Thirdly, read depths of pathogens varied largely and possible amplification bias by PCR during the library preparation and the inherent variation between next generation sequencing runs may have also affected the results. Another probability is the presence of viruses as a causative organism in endophthalmitis, as has been reported by Lee *et al*.^6^ has not been investigated in this study. Additionally, the true positivity of these culture negative samples could only be confirmed by Sanger Sequencing of DNA products in only five of the fourteen samples which revealed sequences matching *Staphylococcus* sp. in two, 1 matching *Streptococcus* sp., and 2 matching *Bacillus* sp. Additionally in some samples, where the vitreous samples showed polymicrobial organisms, validation of NGS data by Sanger sequencing was not possible as ambiguous electropherograms hindered identification of bacteria or fungus. Sequencing of the remaining samples could not be carried out due to exhaustion of the sample after routine microbiological and NGS analysis. The pathologic significance of our study in the setting of culture negative endophthalmitis remains to be elucidated. Until now clinicians have had recourse to only culture reports for the management of patients of endophthalmitis. Our data shows that there is much more happening in the vitreous than what is visible in culture. This study makes a case for considering the role of DNA of organisms in vitreous in the pathogenesis of endophthalmitis and further studies would show the validity of this approach.

In conclusion, our study provides proof of concept that NGS is a powerful approach to identify DNA of pathogens in the vitreous fluid of patients with endophthalmitis and is complementary to microbiological culture based approaches. It also has the potential to revolutionize the diagnosis of culture negative endophthalmitis in tertiary care hospitals with advanced molecular biology facilities and surveillance studies mapping endophthalmitis incidences.

## Methods

### Patients

This was a prospective pilot study that was approved by the L V Prasad Eye Institute - Institutional Review Board (LEC 11-16-112) and the research adhered to the tenets of the Declaration of Helsinki. The study included 34 patients who presented to the Retina clinic, were diagnosed clinically as infectious endophthalmitis and underwent a diagnostic vitreous biopsy/vitrectomy between November 2016 and January 2017. Sample size was limited by the cost of next generation sequencing but was chosen to provide at least 50% culture-negative samples for analysis. As a control group, 30 consecutive patients with uninflamed eyes undergoing vitrectomy for non-infectious retinal disorders (diabetic retinopathy or macular hole) were consented for vitreous tap done during the procedure. Following informed consent from all subjects, clinical details were collected which included demography, cause and duration of symptoms, presenting and final visual acuity along with surgical interventions. Vitreous fluid was collected aseptically from all cases and sent immediately for routine microbiological processing.

### Microbiology

The microbiological work up included direct microscopy and culture as described previously^4^. A portion of the sample, approx. 200μl was stored at −20 °C for molecular tests, however in case of control samples, all of it was kept for molecular analysis. In case of positive cultures, the bacteria were further subjected to identification using Vitek 2 Compact (bioMérieux, France) automated identification system and fungus was identified after observation of colony characteristics and spores on lactophenol cotton blue wet mount.

### DNA purification

Genomic DNA was isolated from vitreous or aqueous fluid using the QIAamp DNA minikit (Qiagen, Germany).

### PCR amplification, Illumina library preparation and amplicon sequencing of the V3-V4 region of 16S rRNA gene and ITS region

The V3–V4 hypervariable region of the 16S rRNA gene which is nearly 465 bp was amplified with the universal primers reported by Klindworth *et al*.^11^, and fused with Illumina adapter overhang nucleotide sequences. The V3–V4 region was amplified using primers 5′-CCTACGGGNGGCWGCAG-3′ and 5′-GACTACHVGGGTATCTAATCC-3′. The thermal profile for amplification comprised an initial denaturation for 5 min at 95 °C, followed by 35 cycles of denaturation at 95 °C for 50 s, annealing at 53 °C for 30 s and elongation at 72 °C for 1 min. A final elongation for 10 min at 72 °C was provided. Similarly the internal transcribed spacer (ITS) region, roughly 650-bp region of the nuclear ribosomal repeat unit was amplified using primers reported by Kumar and Shukla^12^ of the ITS2 region, ITS3 5′- GCATCGATGAAGAACGCAGC-3′) and ITS4 (5′- TCCTCCGCTTATTGATATGC-3′). The thermal profile for amplification comprised an initial denaturation for 10 min at 96 °C, followed by 40 cycles of denaturation at 95 °C for 60 s, annealing at 56 °C for 60 s and elongation at 72 °C for 60 s. A final elongation for 10 min at 72 °C was provided. The amplicon libraries were prepared using Nextera XT Index Kit (Illumina inc.) as per the 16S Metagenomic Sequencing Library preparation protocol (Part #15044223 Rev. B). The amplicons with the Illumina adapters were amplified using i5 and i7 primers that add multiplexing index sequences as well as common adapters required for cluster generation (P5 and P7) as per the standard Illumina protocol. The amplicon libraries were purified by 1X AMpureXP beads and checked with Agilent High Sensitivity (HS) chip on Bioanalyzer 2100 and quantified with fluorometer using Qubit dsDNA HS Assay kit (Life Technologies, India). The libraries were sequenced using Illumina HiSeq 2 × 250 bp chemistry with paired-end protocol at at SciGenomics Pvt. Ltd., Cochin.

### Taxonomic analysis by NGS

After trimming the unwanted sequences from original paired-end data a consensus V3-V4 region sequence was constructed using FLASH program. Low quality reads were filtered using PRINSEQ (PReprocessing and INformation of SEQuences) tool and reads with an average quality score of ≥30 were selected for further analysis using Quantitative Insights Into Microbial Ecology (QIIME) suite^13^. After removal of chimeric sequences using Usearch61, pre-processed reads from all samples were pooled and clustered into Operational Taxonomic Units (OTUs) based on their sequence similarity using Uclust program (similarity cutoff = 0.97). OTUs with only one read in it were identified as Singletons OTUs and were removed and the remaining OTUs were selected for further analysis. Reads/OTUs (reference-OTUs) which matched the reference OTU database were assigned taxonomic lineages as provided in the database, whereas, taxonomic assignments for *de novo* clustered OTUs were obtained using the MOTHUR pipeline (version v.1.29.2). For this purpose, representative sequences from each of the denovo-OTUs were provided as input to the Wang Classifier^14^ (bootstrap threshold of 80%).

The Greengenes (V 13.8) and Unite (V 12.11) databases were used as reference for bacterial and fungal analyses respectively. The obtained OTU tables were used for all further analyses. Sparse OTUs (with <0.001% reads) of the total number of high quality reads were removed and taxonomic relative abundance profiles at genera level were generated based on OTU annotation. The sequences that do not have any alignment against taxonomic database were categorized as “Unknown or Unassigned”.

All data generated or analysed during this study are included in this published article or as Supplementary Data (Tables 1 and 2).

### Identification of ambiguous and contaminant DNA sequences

After sequencing of DNA extraction (blank) control samples we obtained reads, albeit at a lower frequency compared to the patient specimens. The contaminant taxa were removed from the datasets of all endophthalmitis patients and only those that were detected at a higher abundance in these samples and the reads from the pathogens were reported. We did not include species that were not usually reported as causing endophthalmitis which included *Dermacoccus*, *Streptophyta*, *Paracoccus*, *Thermomicrobia* and *Methylobacterium* sp.

### Assay Performance and Statistics

The sensitivity and specificity of NGS as a diagnostic tool in endophthalmitis was compared to culture and clinical diagnosis. Additionally for OTU differential abundance testing between groups Mann-Whitney- Wilcoxon test was carried out between culture positive and culture negative cases (Benjamini Hochberg (BH) corrected P < 0.05).

## Supplementary information

Dataset 1

Dataset 2

Supplementary Table 3
