# Supplementary material for: New insights into culture negative endophthalmitis by unbiased next generation sequencing
Source: Sci Rep. 2019 Jan 29;9:844. doi: 10.1038/s41598-018-37502-w (PMC6351655; doi:10.1038/s41598-018-37502-w)
Supplement: Supplementary file 3 — Supplementary Table 3 [file 41598_2018_37502_MOESM3_ESM.pdf]

New insights into culture negative endophthalmitis by unbiased next generation sequencing

[1]Dhanshree Deshmukh, [1]Joveeta Joseph*, [1]Moumita Chakrabarti, [1]Savitri Sharma, [1]Rajagopalaboopathi Jayasudha, [1]Kalyana C Sama, [1]Bhavani Sontam, [2]Mudit Tyagi, [2]Raja Narayanan, [1]S Shivaji

[1]Jhaveri Microbiology Centre, Brien Holden Eye Research Centre, L. V. Prasad Eye Institute, Hyderabad, Telangana, India, [2] Smt. Kannuri Santhamma Centre for vitreoretinal diseases. L V Prasad Eye Institute

Supplementary Table 3: Statistical comparison of bacterial genera abundance between culture positive and culture negative endophthalmitis cases

Bacterial genera exhibiting significant (BH corrected P < 0.05) differential abundance across culture positive and culture negative endophthalmitis cases

**Genus**	**p value (Wilcoxon test)**	**BH corrected p value**
Acinetobacter	0.353	0.53
Actinomycetales	0.353	0.53
Bacillus	0.165	0.53
Corynebacterium	0.353	0.53
Brevibacterium	0.559	0.789
Enterobacteriaceae	0.203	0.53
Erwinia	0.165	0.53
Gemella	0.638	0.851
Haemophilus	1	1
Klebsiella	0.038	0.344
Microbacterium	1	1
Micrococcus	0.353	0.53
Moraxellaceae	1	1
Morganella	1	1
Planococcaceae	0.97	1
Pseudomonas	1	1
Serratia	0.038	0.344
Sphingomonas	0.3	0.53
Staphylococcus	0.319	0.53
Streptococcus	0.043	0.344
Williamsia	0.096	0.53
Salmonella	0.353	0.53
Stenoptrophomonas	0.353	0.53
Unidentified	0.353	0.53
